# Aquaporin 3 (AQP3) participates in the cytotoxic response to nucleoside-derived drugs

**DOI:** 10.1186/1471-2407-12-434

**Published:** 2012-09-27

**Authors:** Sandra Pérez-Torras, F Javier Casado, Marçal Pastor-Anglada

**Affiliations:** 1 Departament de Bioquímica i Biologia Molecular, Facultat de Biologia, Institut de Biomedicina de la Universitat de Barcelona (IBUB), Universitat de Barcelona, and Centro de Investigación Biomédica en Red – Enfermedades Hepáticas y Digestivas (CIBER EHD), Diagonal 645, 08028, Barcelona, Spain; 2 Present address: Centro Nacional de Investigaciones Cardiovasculares (CNIC), Madrid, Spain; 3 Present address: Signal Transduction Laboratory, Cancer Research UK London Research Institute, London, UK

## Abstract

**Background:**

Nucleoside analogs used in the chemotherapy of solid tumors, such as the capecitabine catabolite 5^′^-deoxy-5-fluorouridine (5^′^-DFUR) trigger a transcriptomic response that involves the aquaglyceroporin aquaporin 3 along with other p53-dependent genes. Here, we examined whether up-regulation of aquaporin 3 (AQP3) mRNA in cancer cells treated with 5^′^-DFUR represents a collateral transcriptomic effect of the drug, or conversely, AQP3 participates in the activity of genotoxic agents.

**Methods:**

The role of AQP3 in cell volume increase, cytotoxicity and cell cycle arrest was analyzed using loss-of-function approaches.

**Results:**

5^′^-DFUR and gemcitabine, but not cisplatin, stimulated AQP3 expression and cell volume, which was partially and significantly blocked by knockdown of AQP3. Moreover, AQP3 siRNA significantly blocked other effects of nucleoside analogs, including G_1_/S cell cycle arrest, p21 and FAS up-regulation, and cell growth inhibition. Short incubations with 5-fluorouracil (5-FU) also induced AQP3 expression and increased cell volume, and the inhibition of AQP3 expression significantly blocked growth inhibition triggered by this drug. To further establish whether AQP3 induction is related to cell cycle arrest and apoptosis, cells were exposed to long incubations with escalating doses of 5-FU. AQP3 was highly up-regulated at doses associated with cell cycle arrest, whereas at doses promoting apoptosis induction of AQP3 mRNA expression was reduced.

**Conclusions:**

Based on the results, we propose that the aquaglyceroporin AQP3 is required for cytotoxic activity of 5’-DFUR and gemcitabine in the breast cancer cell line MCF7 and the colon adenocarcinoma cell line HT29, and is implicated in cell volume increase and cell cycle arrest.

## Background

Nucleoside analogs are currently employed in cancer treatment. These compounds exert cytotoxic effects by interfering with the uptake and metabolism of their natural counterparts. They trigger transcriptomic responses preferentially encompassing up-regulation of a set of genes implicated in cell cycle regulation and apoptosis along with other genes of undefined function in cancer chemotherapy [1-4]. Among these “non-anticipated” genes, we identified aquaporin 3 (AQP3) [4]. AQP3-related mRNA levels dramatically increased (8-fold) after treatment of MCF7 breast cancer cells with the capecitabine catabolite, 5^′^-deoxy-5-fluorouridine (5^′^-DFUR), a direct precursor of 5-fluorouracil (5-FU). Treatment of these cells with the human Equilibrative Nucleoside Transporter-1 (hENT1) inhibitor, NBTI, led to significant resistance to 5^′^-DFUR, which was associated with a marked decrease in AQP3 up-regulation. Thus, it appears that changes in AQP3-related mRNA levels parallel the cytotoxic effects of nucleoside derivatives on breast cancer cells.

Aquaporins (AQPs) are integral membrane proteins implicated in the selective transport of water across the plasma membrane. A subset of the AQP family that includes AQP3 also mediates glycerol uptake. Accordingly, these proteins are designated aquaglyceroporins [5-7]. When AQP3 was initially identified as putative drug target, limited information was available on the role of this protein family in cancer. Recent evidence suggests that selective AQP participate in angiogenesis, cell migration and metastasis (reviewed by [8]). AQP1-null mice display reduced tumor growth after subcutaneous implantation of melanoma cells, which is associated with reduced endothelial cell migration and angiogenesis [9]. Moreover, AQP1 expression promotes tumor cell extravasation and metastasis [10]. AQP3 has been implicated in skin tumorigenesis. AQP3-null mice are resistant to the development of skin tumors, while skin squamous cell carcinomas overexpress this protein [11]. Clinical data from a number of studies provide evidence for the heterogeneous expression of different AQP family members in solid tumors, and in most cases, AQP overexpression [12-15].

The possibility that a particular AQP gene member is implicated in the chemotherapeutic response to antitumor agents has not been addressed. Moreover, previous studies reporting acute AQP3 up-regulation following nucleoside-derived drug treatment in cultured cancer cells do not provide insights into whether changes in the AQP3-related mRNA level represent a collateral effect of treatment or, on the contrary, it participates in drug response, either by promoting it or by acting as a resistance gene. In this study, we address whether AQP3 is implicated in drug responses by monitoring the effects of gene silencing on expression patterns of nucleoside analogs-induced target genes, cell cycle progression, and cell growth in the breast cancer cell line MCF7 and the colon adenocarcinoma cell line HT29.

## Methods

### Reagents

5^′^-DFUR, 5-fluorouracil, cisplatin (*cis*-diaminedichloroplatinum or *cis*-DDP) and propidium iodide were purchased from Sigma-Aldrich (Saint Louis, MO, USA). Gemcitabine (2^′^,2^′^-difluorodeoxycytidine, dFdC, Gemzar®) was obtained from Eli Lilly and Company (Indianapolis, IN, USA).

### Cell culture and treatments

The human colorectal carcinoma cell line HT29 (HTB-38, ATCC-LGC Promochem Partnership, USA) and two human breast carcinomas cell lines, MCF7 (HTB-22, ATCC-LGC Promochem Partnership, USA) and MDA-MB-468 (HTB-132, ATCC-LGC Promochem Partnership, USA) were purchased from the American Type Culture Collection with the indicated references. MCF7 and MDA-MB-468 cell lines are characterized by the fact that the former expresses the estrogen and progesterone receptors whereas the latter is negative for both. NP-29 cells were derived from human pancreatic adenocarcinomas, which had been perpetuated as xenografts in nude mice and further characterized for different oncogene and tumor suppressor profiles [16]. MCF7 and HT29 cells were cultured in Dulbecco’s Modified Eagle Medium (DMEM) supplemented with 10% fetal bovine serum (GIBCO-BRL, Grand Island, NY, USA), 2 mM glutamine, and a mixture of antibiotics (100 U penicillin, 0.1 mg/ml streptomycin and 0.25 μg/ml fungizone). The MDA-MB-468 cell line was maintained in DMEM and F12 mixture (1:1) supplemented with 10% fetal bovine serum, 2 mM glutamine and 100U penicillin, 0.1 mg/ml streptomycin. NP-29 cells were maintained in DMEM and F12 mixture (1:1) supplemented with 5% fetal bovine serum, 2 mM glutamine and 100U penicillin, 0.1 mg/ml streptomycin. Cells were maintained as monolayer cultures at 37°C in an atmosphere containing 5% CO_2_, and subcultured by trypsinization every 4–5 days. *Mycoplasma* test assays, verification of morphology and growth curve analysis were performed as a routine protocol for all of them. Cells were treated 24 h after seeding at 20 000 cells/cm^2^. Cultures were exposed to drugs for 90 min (5^′^-DFUR: 250 μM; 5-FU: 250 μM; gemcitabine: 100 nM for MCF7, 250 nM for MDA-MB-468 and NP-29 and 50 μM for HT29; cisplatin: 50 μM), and measurements performed at 24 or 48 h after drug addition. Drug concentrations were chosen based upon the EC_75_ values calculated from MTT cell viability assays, as previously described [4,17]. The choice of 90 min was based upon the need to highlight the role transport processes play in drug action but, more importantly, to better mimic the in vivo exposure time to the drug, which is far less shorter than the “classical” cytotoxicity assays in which cells are exposed to drugs for 24, 48, and even 72 hours.

### RNA isolation and quantitative RT-PCR

Isolation of mRNA was performed after treatment using the SV Total RNA Isolation System (Promega Biotech, Madison, WI, USA), following the manufacturer’s protocol. Total DNase-treated RNA (1 μg) was used to generate cDNA using M-MLV Reverse Transcriptase (Promega Biotech) and random hexamers (Amersham Pharmacia, Buckinghamshire, UK) for reverse transcription. Quantitative real-time PCR was performed with the ABI PRISM 7700 Sequence Detection System (Applied Biosystems, Foster City, CA) using the manufacturer’s recommendations. Assays-on-Demand Taqman probes (Applied Biosystems) for AQP3, CDKN1A/p21, TNFRSF6/FAS and GAPDH were employed (Hs00185020_m1, Hs00355782_m1, Hs00163653_m1 and 4310884E, respectively). Relative quantification of gene expression was performed as described in the TaqMan user manual with GAPDH as an internal control.

### Measurement of cell volume and cell counting

Cells were plated in 24-well culture plates. After 24 h, cells were treated for 90 min with different genotoxic agents. Cultures were allowed to proceed for 48 h. The cell culture was washed and the remaining cells were trypsinized and collected in culture medium. Cell volume and number were measured using a cell counter Coulter Multisizer (Beckman Coulter, Inc., Fullerton, CA) or Quanta SC flow cytometer (Beckman Coulter). The population of viable cells was discriminated by size and the number of cells was calculated as a percentage by comparing the cell number from treated cultures with that from cultures not exposed to cytotoxic drugs.

### Transfection with small interfering RNA (siRNA) for AQP3

AQP3 siRNA (ID: 147362) was purchased from Ambion (Austin, TX, USA). Silencer® Negative Control siRNA #1 (Ambion) was employed as the negative control to ensure silencing specificity in all the experiments.

Transfection of cells with 20–25 nM (MCF7) or 200 nM (HT29) of siRNA was performed using Lipofectamine 2000® (Invitrogen, Carlsbad, CA, USA), according to the manufacturer’s recommendations. Transfection efficiency was measured using AQP3 siRNA (ID: 147362) labeled with FAM (6-carboxy-fluorescein) and a Beckman Coulter flow cytometer (Fullerton, CA). Depletion of AQP3 expression following siRNA transfection was confirmed by real-time RT-PCR, as described above.

### Cell cycle analysis

At 48 h after treatment, cells were collected by centrifugation at 1200 g for 4 min and fixed in cold 70% ethanol. After 24 h, cells were washed and resuspended in 0.5 ml of PBS containing RNase (10 μg/ml). Flow cytometry analysis was performed within 1 h after the addition of propidium iodide (0.1 mg/ml) at room temperature using a Coulter XL (Beckman Coulter).

### Western blot analysis

Cells were lysed in a RIPA buffer containing 1% Complete Mini protease inhibitors (Roche, Mannheim Germany). Protein concentration was determined by the Bradford assay (Bio-Rad, Hercules, CA) and 30 μg of total protein were resolved by electrophoresis on 12% SDS-PAGE gels and transferred to PVDF membranes by standard methods. Membranes were immunoblotted with anti-p21 (Santa Cruz, Santa Cruz, CA), anti-Fas (Roche, Mannheim, Germany) and anti-tubulin (Sigma, St Louis, Mo) and the corresponding secondary antibodies, horseradish peroxidase (HRP)-conjugated antibodies (Bio-Rad, Hercules, CA). Antibody labeling was detected using the chemiluminiscence detection kit (Biological Industries, Israel).

### Apoptosis detection

Apoptosis was measured using the Annexin V-FITC Apoptosis Detection Kit I (BD Biosciences, San Diego, CA). Cells were harvested by centrifugation (including detached cells) 48 h after treatment with increasing doses of 5-fluorouracil (5–500 μM), washed twice in PBS, and pelleted again. They were resuspended at 10^6^ cells/ml in binding buffer, 100 μl of cells were stained with 5 μl Annexin-V and 5 μl propidium iodide, and incubated in the dark for 15 min at room temperature, as recommended by the manufacturer. Following the addition of 400 μl binding buffer, cells were processed within 1 h using the FACScan flow cytometer Coulter XL (Beckman Coulter).

### Statistical analysis

The paired or unpaired Student’s t-test was used to compare experimental data. Analysis was performed using GraphPad Prism software (GraphPad Software, Inc., San Diego, CA).

## Results

### Up-regulation of AQP3 expression by genotoxic agents

**Figure 1:**
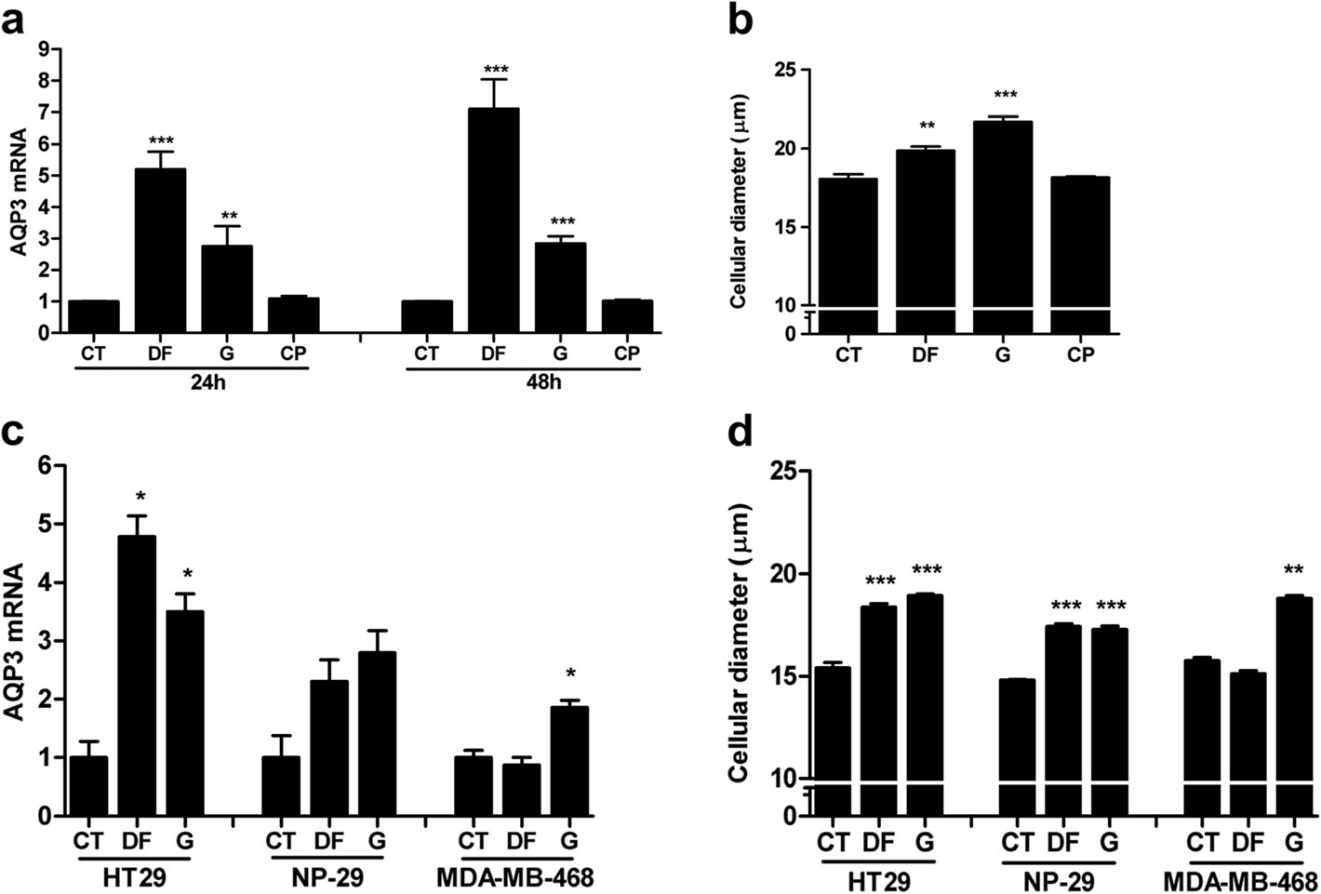
**Effect of genotoxic drugs on AQP3 expression and cell volume.** MCF7 (**a**) or HT29, NP-29 and MDA-MB-468 (**c**) cells were incubated for 90 minutes (5^′^-DFUR (DF): 250 μM; gemcitabine (G): 100 nM for MCF7, 250 nM for NP-29 and MDA-MB-468 and 50 μM for HT29; cisplatin (CP): 50 μM) and mRNA was isolated at 24 (a) or 48 hours (a, c). Real-time RT-PCR analysis for AQP3 was performed using GAPDH as endogenous control. Data are calculated as arbitrary units relative to untreated cells (CT) as a reference. Results are the mean ± SE of three to six independent experiments measured in duplicate. At 48 hours after 90 min exposure to the genotoxic drugs, MCF7 (**b**) or HT29, NP-29 and MDA-MB-468 (**d**) cells were collected and volumes were measured as cell diameters (μm). Results are the mean ± SE of three to four independent experiments measured in triplicate. Statistical significance was assessed with the Student’s t test (* p < 0.05; ** p < 0.01; *** p < 0.001).

AQP3 was previously identified as an up-regulated gene in 5^′^-DFUR-treated MCF7 cells using cDNA microarray experiments. To further determine whether up-regulation is specific in response to this particular agent or additionally induced by other genotoxic drugs MCF7 cells were exposed for 90 min to 250 μM 5^′^-DFUR, 100 nM gemcitabine or 50 μM cisplatin, and AQP3 mRNA levels were analyzed by RT-PCR after 24 and 48 h of treatment (Figure 1a). Drug concentrations were selected based on previously calculated EC_75_ values using MTT cell viability assays. Both nucleoside-derived drugs, 5^′^-DFUR and gemcitabine enhanced AQP3-related mRNA levels at the time-points assayed (24 and 48 h), albeit at different magnitudes (5-7-fold by 5^′^-DFUR vs 3-fold by gemcitabine). Interestingly, the alkylating drug cisplatin did not affect the AQP3 mRNA level.

Since AQP3 functions as a water channel, we determined whether induction of the gene is associated with the changes in cell volume after drug treatment. Accordingly, cellular diameter was measured under different treatment conditions, as shown in Figure 1b. Consistent with AQP3 mRNA data, 5^′^-DFUR and gemcitabine, but not cisplatin, induced a significant increase in cell diameter in MCF7 cells, although in this case, the magnitude of the effect of gemcitabine was higher than that of 5^′^-DFUR.

In order to elucidate if this effect could be extended to other cancer cells, effect of 5^′^-DFUR and gemcitabine treatment on AQP3 expression and cell volume were tested in the colon carcinoma cell line HT29, the pancreatic cancer cell line NP-29 and the ER/PR negative breast cancer derived MDA-MB-468. Cells were exposed for 90 min to 5^′^-DFUR or gemcitabine and AQP3 mRNA levels analyzed by RT-PCR after 48 h of treatment (Figure 1c). Drug concentrations were selected based on previously calculated EC_75_ values (5^′^-DFUR: 250 μM; gemcitabine: 250 nM for NP-29 and MDA-MB-468 and 50 μM for HT29). Similarly to MCF7, both nucleoside-derived drugs, 5^′^-DFUR and gemcitabine, enhanced AQP3-related mRNA levels in HT29 and NP-29 albeit at different magnitudes, and gemcitabine also induced an increase in the expression of AQP3 in the MDA-MB-468 cell line. In the same way, the colon cancer cell line HT29 and the pancreatic cancer cell line NP-29 showed an increase in cell diameter after treatment with both nucleoside analog drugs and MDA-MB-468 only exhibited an increased cell volume after gemcitabine treatment (Figure 1d).

### AQP3 knockdown suppresses the increased cell volume and cytotoxicity induced by nucleoside analogs

To establish the specific role of AQP3 in cellular responses to nucleoside-derived drugs, we examined the effects of inhibiting AQP3 expression using siRNA. Transfection of cells with AQP3 siRNA resulted in 75% and 20% reduction in the AQP3-related mRNA levels in MCF7 and HT29 cells respectively (data not shown).

Transfection efficiency, measured using FAM (6-carboxy-fluorescein)-labeled AQP3 siRNA was approximately 75% in MCF7 cells and 55% in HT29 cells. Moreover, AQP3 mRNA silencing lasted for 96 hours since transfection, being able to block the up-regulation of AQP3 expression induced by 5^′^-DFUR treatment (data not shown).

**Figure 2:**
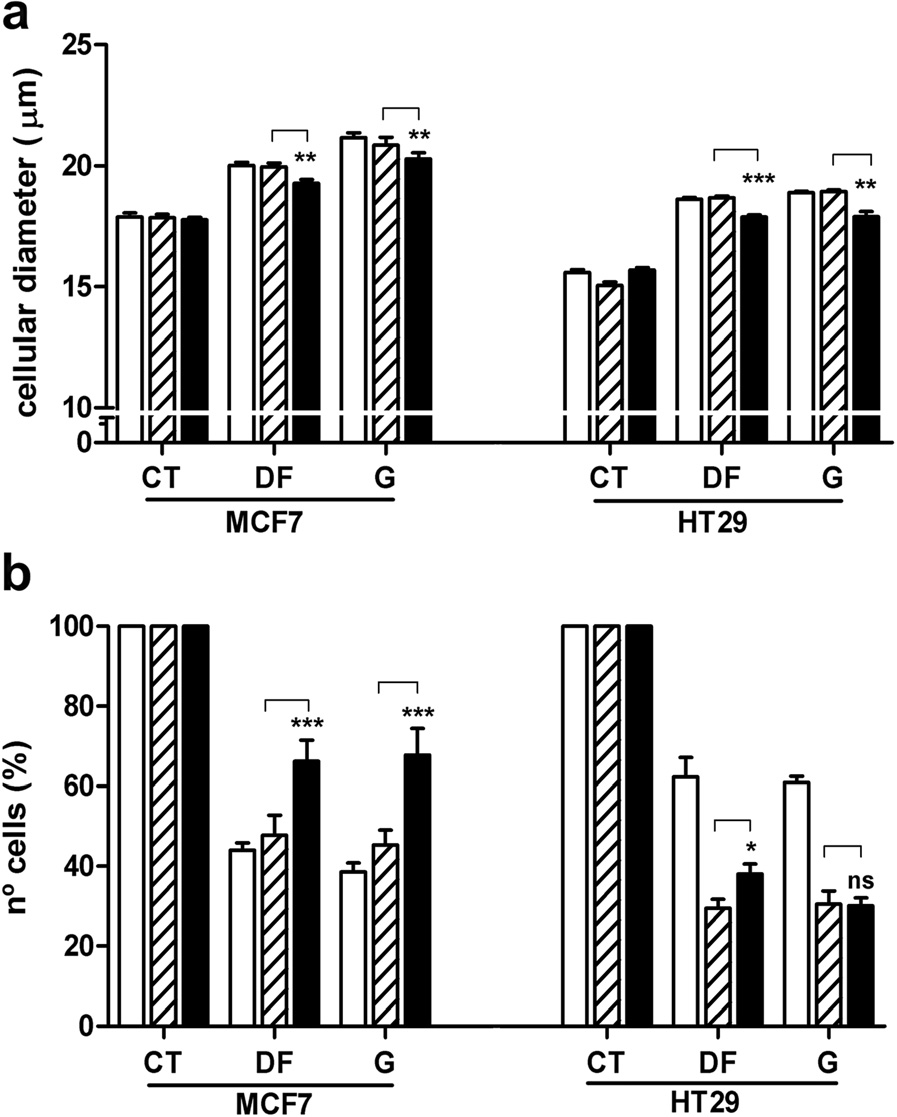
**Effects of AQP3 silencing on cell volume changes and inhibition of cell growth induced by nucleoside analogs.** Non-transfected (white), negative control siRNA (dashed) or AQP3-siRNA (black) transfected MCF7 or HT29 cells were treated for 90 min with 250 μM 5^′^-DFUR or 100 nM gemcitabine for MCF7 and 50 μM gemcitabine for HT29. After 48 hours cells were trypsinized and diameter was measured as an estimation of volume changes (**a**) along with number of cells (**b**) using a cell counter. Cell viability is calculated as a percentage basis in relation to untreated cells as a reference. Results are presented as means ± SE of six independent experiments measured in triplicate. Statistical significance was assessed with the paired Student’s t test (* p < 0.05; ** p < 0.01; *** p < 0.001).

To assess the putative role of AQP3 in cell volume regulation in response to genotoxic agents, we measured changes in the cell diameter after nucleoside analog treatment in non-transfected, negative control siRNA-transfected and AQP3 siRNA-transfected cells. Cells were incubated for 90 min with 5^′^-DFUR or gemcitabine (5^′^-DFUR: 250 μM; gemcitabine: 100 nM for MCF7 and 50 μM for HT29), and cell diameters measured after 48 h (Figure 2a). As shown previously, both drugs induced a marked increase in cell diameter. Inhibition of AQP3 expression significantly reduced but did not fully prevent the increase in cell volume triggered by the nucleoside-derived drugs in MCF7 and HT29 cells. Both nucleosides additionally exerted dramatic effects on cell viability as determined by measuring the number of cells after 48 h of treatment (Figure 2b). Similarly to cell volume changes, AQP3 silencing resulted in significant reversion of nucleoside-induced cell growth inhibition in the breast cancer cell line MCF7, and to a lesser extent in the colon cancer cell line HT29 after treatment with 5^′^-DFUR. However, the cell growth arrest induced by gemcitabine in HT29 was not blocked by the inhibition of AQP3 expression.

Interestingly, similar results were initially obtained upon blocking the activity of AQP3 with CuSO_4_ in MCF7 cells (data not shown). Copper salts are effective AQP3 inhibitors [18,19] but also can display toxicity, and independently exert a variety of effects on cell responses to DNA damage. Thus, inhibition of AQP3 activity supports the data obtained when silencing AQP3 expression.

### AQP3 silencing partially reverses cell cycle arrest triggered by nucleoside-derived drugs and up-regulation of transcriptional targets

**Figure 3:**
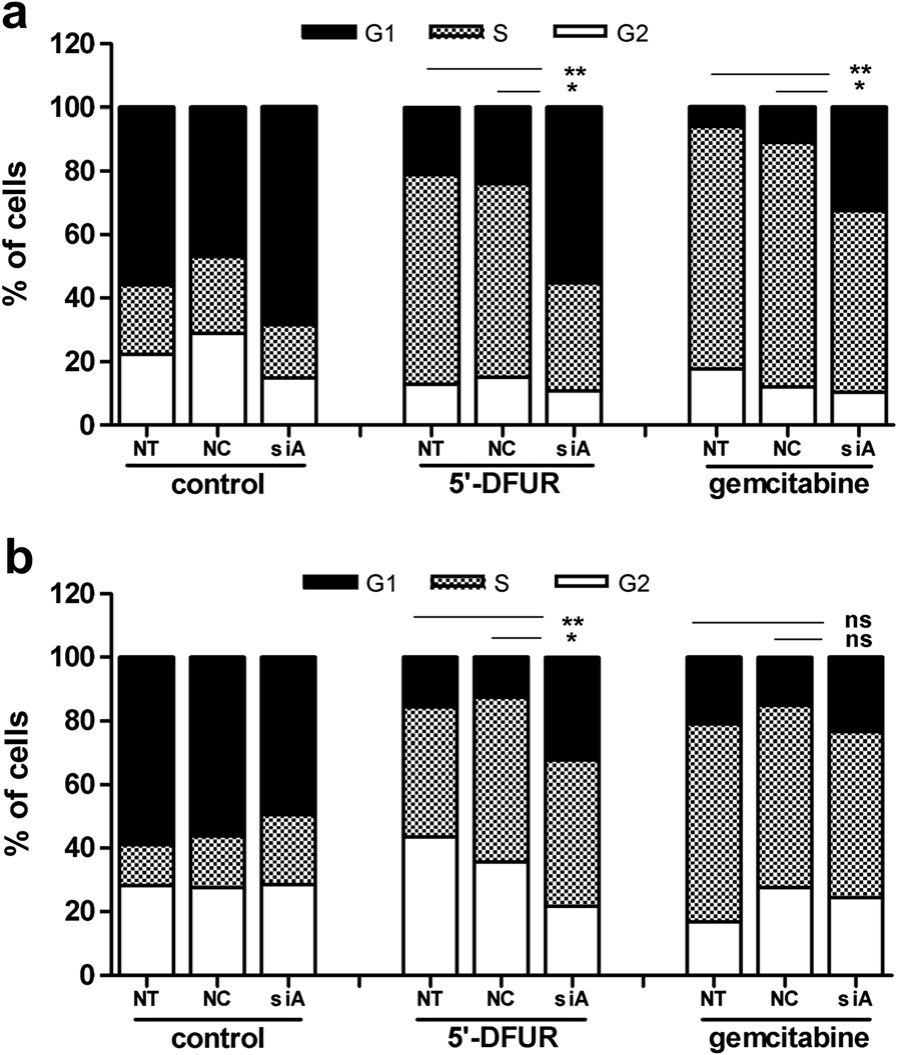
**Effects of genotoxic agents on the cell cycle.** 48 hours after 90 min treatment with genotoxic drugs (250 μM 5^′^-DFUR, 100 nM gemcitabine for MCF7 and 50 μM gemcitabine for HT29), non-transfected (NT), negative control siRNA (NC) or AQP3-siRNA (siA) transfected MCF7 (**a**) or HT29 (**b**) cells were stained with propidium iodide, and cell cycle was analyzed with a flow cytometer. Results are presented as means of four to six independent experiments. Statistical significance was assessed with the Student’s t test (* p < 0.05; ** p < 0.01).

Treatment of cells with 5^′^-DFUR and gemcitabine induced cell cycle arrest at the G_1_-S phase in MCF7 cells (Figure 3a), whereas cisplatin promoted accumulation of cells at the S-G_2_ phase (data not shown), fact that had previously been reported [20]. Interestingly, AQP3 siRNA significantly blocked cell cycle arrest induced by both nucleoside-analogs in MCF7 cells (Figure 3a). Similarly to the reversion of cell growth inhibition in HT29 cell line (Figure 2b), only the cell cycle arrest triggered by 5^′^-DFUR was reversed, but not the one triggered by gemcitabine **(**Figure 3b). To eliminate the possibility that cell cycle-dependent regulation of AQP3 expression interferes with these phenomena, MCF7 cells were synchronized by serum depletion, and AQP3-related mRNA levels analyzed during cell cycle progression. Under these conditions, we observed no differences in AQP3 mRNA levels (data not shown).

**Figure 4:**
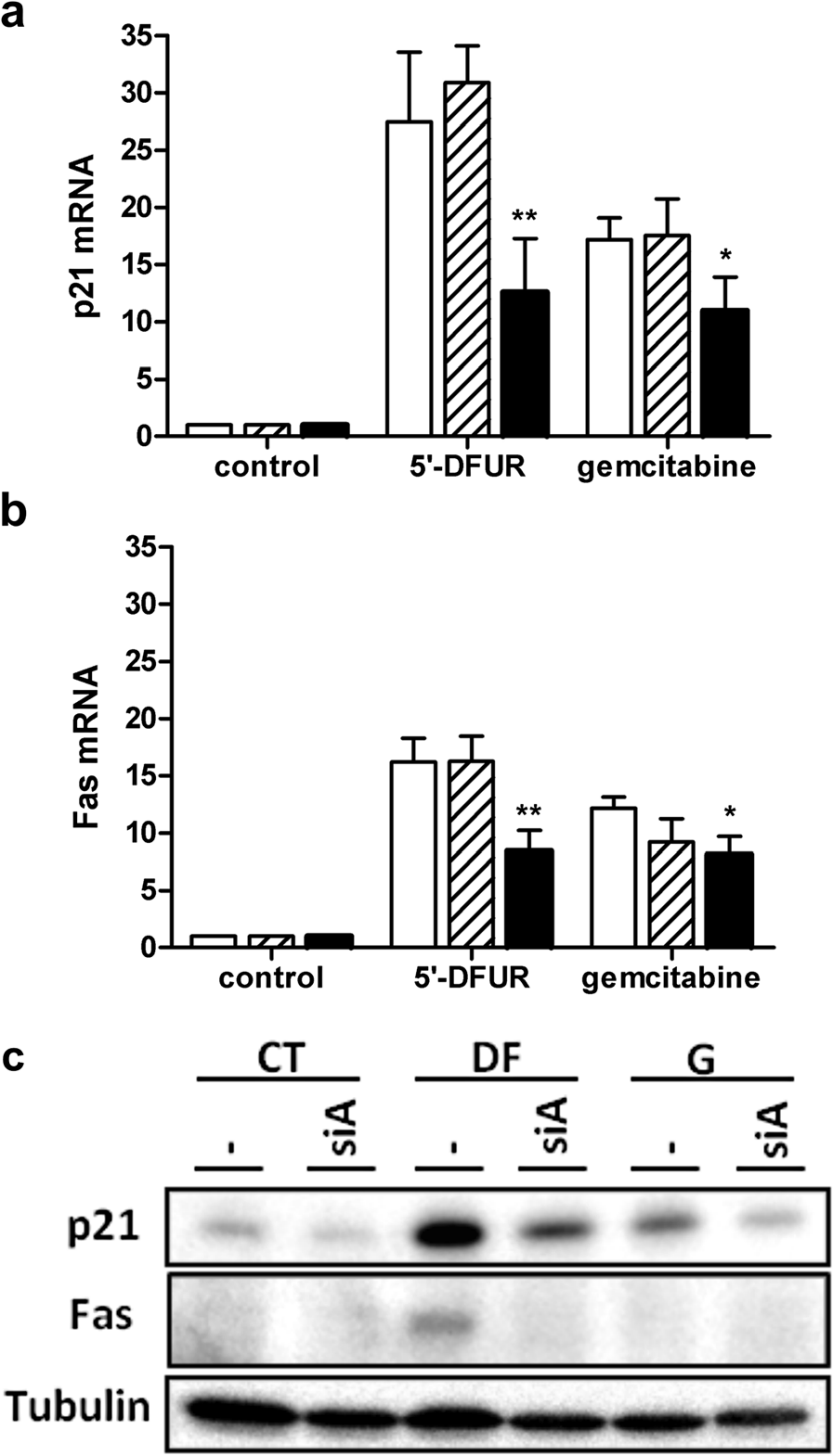
**Inhibition of transcriptional targets of 5’-DFUR and gemcitabine by AQP3-siRNA.** Non-transfected (white), negative control siRNA (dashed) or AQP3-siRNA (black) transfected MCF7 cells were incubated for 90 minutes with 250 μM 5’-DFUR or 100 nM gemcitabine. After 24 h, RNA was isolated and real-time RT-PCR analysis for p21 and Fas performed (**a** and **b**, respectively). C_T_ values for each gene are normalized to an endogenous reference gene (GAPDH). mRNA levels are calculated in arbitrary units using control values as the reference. Results are presented as means ± SE of four to six independent experiments measured in duplicate. Statistical significance was assessed with the Student’s t test (* p < 0.05; ** p < 0.01). (**c**) Non-transfected or AQP3-siRNA transfected MCF7 cells were treated for 90 minutes with 250 μM 5’-DFUR or 100 nM gemcitabine and Western blot analysis was perfomed for p21, Fas and α-tubulin as a loading control. A representative Western blot is shown.

5^′^-DFUR and gemcitabine up-regulate a variety of genes, generally in a p53-dependent manner [4,17]. We analyzed whether AQP3 knockdown affects the transcriptional response associated with drug treatment in MCF7, cell line in which we observed the clearest effects on cell cycle. Non-transfected, negative control siRNA-transfected or AQP3 siRNA-transfected cells were incubated for 90 min with either 5^′^-DFUR or gemcitabine, and p21 and Fas expression analyzed after 24 h at the mRNA level using real-time PCR (Figure 4a and 4b) or at the protein level by western blot (Figure 4c). Inhibition of AQP3 expression led to partial blockage of the increase in p21 and Fas mRNA levels induced by nucleoside-derived drugs measured at 24 h (Figure 4a and 4b, respectively). AQP3 siRNA-mediated blockage of the increase in p21 and Fas after treatment with 5^′^-DFUR was also confirmed at the protein level (Figure 4c). However, gemcitabine treatment led only to an increase in p21 protein levels, which was reversed by the AQP3 knock-down (Figure 4c).

In terms of previous parameters (cell volume and drug-induced toxicity), similar results were obtained at 24 h upon inhibition of AQP3 activity using CuSO_4_ (data not shown).

### AQP3 silencing reverses cytotoxicity induced by 5-fluorouracil

**Figure 5:**
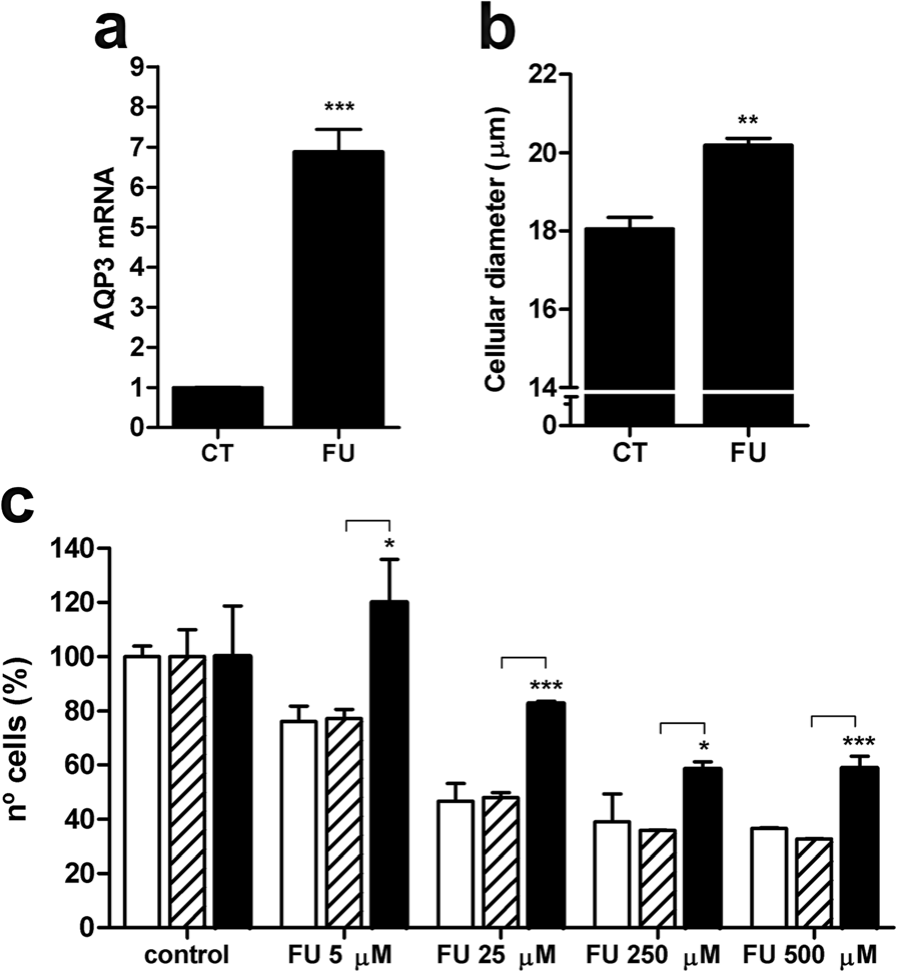
**Effect of 5-fluorouracil on AQP3 expression (a) and cell volume (b) and effect of AQP3 silencing on cell growth inhibition induced by 5-fluorouracil (c).** MCF7 cells were incubated for 90 minutes with 5-FU (FU) 250 μM and mRNA was isolated at 48 hours (**a**). Real-time RT-PCR analysis for AQP3 was performed using GAPDH as endogenous control. Data are calculated as arbitrary units relative to untreated cells (CT) as a reference. Results are the mean ± SE of three independent experiments measured in duplicate. At 48 hours after 90 min exposure to 250 μM 5-FU, MCF7 cells (**b**) were collected and volumes were measured as cell diameters (μm). Results are the mean ± SE of three independent experiments measured in triplicate. (**c**) Non-transfected (white), negative control siRNA (dashed) or AQP3-siRNA (black) transfected MCF7 were treated for 90 min with increasing doses of 5-FU (5 to 500 μM). After 48 h cells were trypsinized and the number of cells was measured using a cell counter. Cell viability is calculated as a percentage in relation to untreated cells as a reference. Results are the mean ± SE of three independent experiments measured in triplicate. Statistical significance was assessed with the Student’s t test (* p < 0.05; ** p < 0.01; *** p < 0.001).

5^′^-DFUR is the immediate precursor of the active fluoropyrimidine 5-fluorouracil (5-FU). To further evaluate if the effects described for 5^′^-DFUR could also be extended to 5-FU, MCF7 cells were incubated for 90 min with 250 μM 5-FU and AQP3 mRNA levels and cell volume were analyzed. Similarly to 5^′^-DFUR effects, AQP3 mRNA expression (Figure 5a) and cell volume (Figure 5b) were increased after 90 min treatment with 5-FU. To analyze the effect of 5-FU on cell viability, we performed a set of experiments in which non-transfected, negative control siRNA-transfected or AQP3 siRNA-transfected cells were treated with different doses of 5FU for 90 min and cell number measured after 48 h. As shown in Figure 5c, escalating doses of 5FU induced a progressive decrease in cell number, which was fully reversed at low 5-FU concentrations (5 μM) or partially but significantly reversed at higher 5-FU concentrations (up to 500 μM) when AQP3 expression was silenced.

### Induction of apoptosis by 5-fluorouracil suppresses the increase in AQP3 expression in MCF7 cells

Under our experimental conditions, 90-minute treatment with either 5^′^-DFUR or 5-FU led to arrest of cell cycle progression at 48 h, but did not ultimately promote apoptosis. Interestingly, longer incubations (48 h) with 5-FU but not with 5^′^-DFUR were able to induce some apoptosis in MCF7 cells. For this reason, long incubations of increasing concentrations of 5-FU were used to further determine whether AQP3 induced by nucleoside analogs plays a role in cell cycle arrest and/or death.

**Figure 6:**
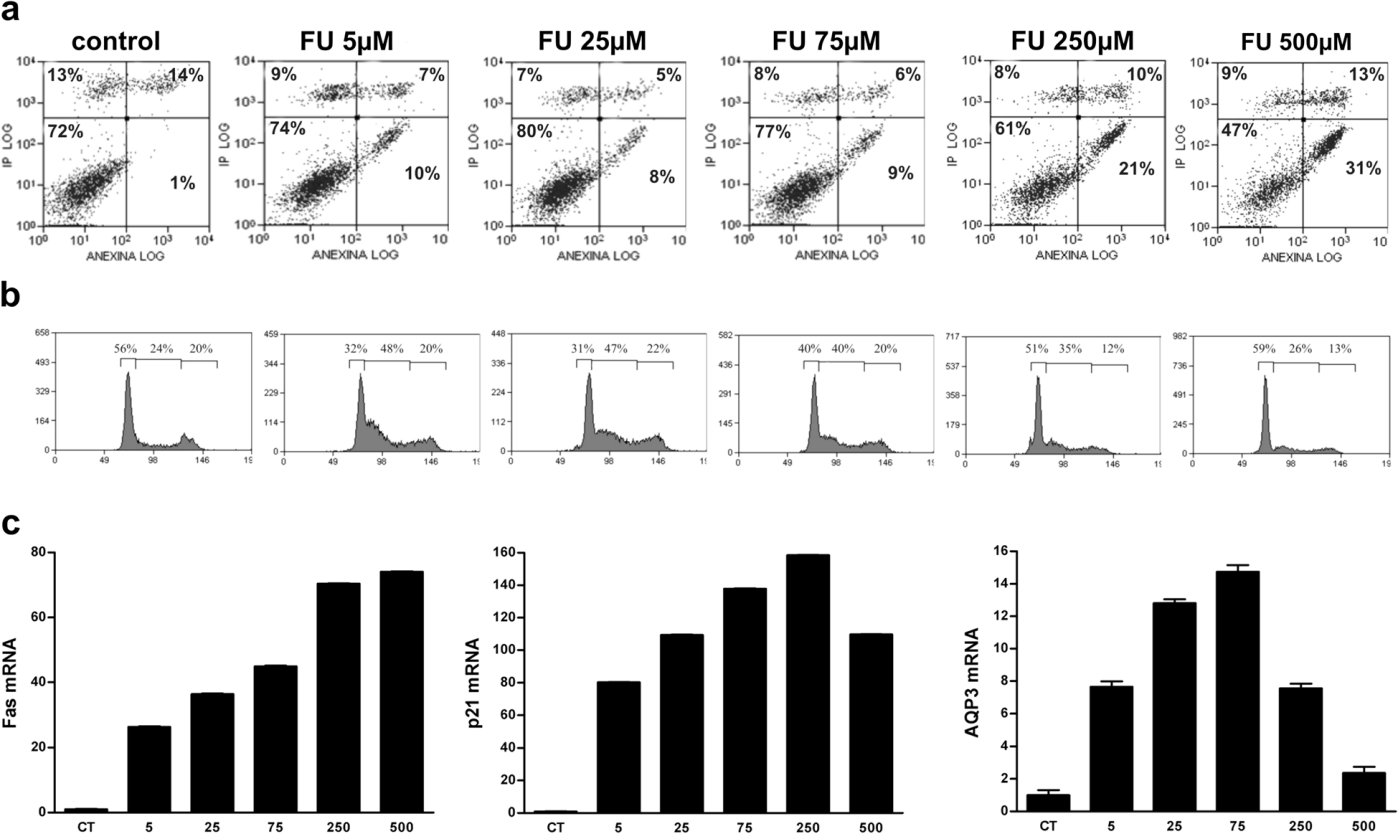
**Effects of 5-FU on apoptosis (a), cell cycle (b), and Fas, p21 and AQP3 mRNA levels (c).** Cells were incubated with increasing doses of 5-FU for 48 hours (**a**) Apoptosis was quantified by double staining with Annexin-V conjugated to FITC and propidium iodide, and analyzed with a FACScan flow cytometer. (**b**) The DNA content was measured using propidium iodide staining and fluorescence-activated cell sorting analysis. (**c**) Real-time RT-PCR for each gene was performed, after RNA isolation using GAPDH as the internal control. mRNA expression levels are presented as arbitrary units in relation to control values as the reference. Results are the mean ± *SE* of a representative experiment measured in triplicate, three independent experiments were performed.

MCF7 cells were treated with increasing doses of 5-FU, and the cell cycle and apoptosis analyzed at 48 h (Figure 6a,b). Treatment with low doses of 5-FU (5, 25 or 75 μM) led to cell cycle arrest at the G_1_-S phase, but not significant cell death. Conversely, upon incubation of cells with 5-FU at high concentrations (250 or 500 μM), increased apoptosis was observed (20-30%) whereas the cell cycle was poorly affected.

The mRNA levels of Fas, p21 and AQP3 were measured under the above conditions (Figure 6c). The peak of FAS-related mRNA levels was achieved at the highest doses of 5-FU, which do not affect cell cycle progression but strongly promote apoptosis. On the other hand, p21-related mRNA amounts linearly increased with 5-FU doses at the lower concentration range (5–75 μM), but were less affected at the highest 5-FU concentration (500 μM). Interestingly, AQP3 expression was dramatically increased at doses associated with cell cycle arrest (5, 25 or 75 μM), whereas upon escalating to concentrations reported to promote apoptosis (250 or 500 μM), the increase in AQP3-related mRNA levels was even reduced, down to near basal levels at 500 μM 5-FU.

## Discussion

High-throughput transcriptomic analysis of anticancer drug activity is a suitable tool to identify novel target genes. However, confirmation that a particular drug-modulated gene specifically contributes to drug response requires detailed analysis similar to that performed for AQP3, a gene up-regulated by the 5-FU precursor and capecitabine catabolite, 5^′^-DFUR, in the breast cancer cell line MCF7 [4].

AQP3 is a broadly expressed aquaglyceroporin found in most epithelia, where it localizes to the basolateral membrane, as well as in several types of nonepithelial cells [21]. The extensive distribution pattern suggests that this water channel protein is a major player in barrier hydration and water and osmolyte homeostasis. AQP3 is a target of aldosterone in the collecting duct [22] and under osmotic control in renal and keratocarcinoma cells, thus presumably contributing to cell volume adaptive regulatory processes [23,24]. While previous studies suggest that changes in cell size associated with cell division are facilitated by increased AQP1 abundance at the plasma membrane [25], our results support a putative role of AQP3 in maintaining or promoting cell swelling induced by nucleoside-derived drugs. Interestingly, AQP3-related mRNA levels were not modified during cell cycle progression, suggesting that the role of the water channel in the increased cell volume is related to drug response. The nucleoside analogs 5^′^-DFUR and gemcitabine triggered G_1_/S cell cycle arrest, but not cisplatin. This DNA alkylating agent appeared to induce S/G_2_ arrest, which did not result in increased cell volume, in contrast to the effects of nucleoside-derived drugs.

Knockdown of AQP3 expression produced a partial but significant reversion of increased cell swelling associated with nucleoside-derived drug treatment, further supporting a role of AQP3 in this process. Nevertheless, the magnitude of cell volume reversion in MCF7 and HT29 (about 25%), even assuming that AQP3 expression is only partially blocked in siRNA-transfected cells, suggests that this water channel protein is not the only contributor to cell swelling associated with drug treatment. Interestingly, under similar conditions, suppression of AQP3 preserved cell growth inhibition to a better extent, and the magnitude of reversion of G_1_/S cell cycle arrest was significantly higher than reversion of cell swelling for 5^′^-DFUR and gemcitabine in MCF7 cells. Furthermore, in spite of achieving only a 20% of AQP3 mRNA knockdown in HT29, AQP3 suppression partially reverted cell cycle arrest and preserved cell growth inhibition in 5^′^-DFUR treated cells. Thus, it is possible that AQP3 plays roles other than those derived from its ability to mediate water transport. In fact, AQP3 plays a variety of roles in cell physiology associated with its ability to take up glycerol. AQP3-deficient mice show defective skin hydration and elasticity, which can be corrected by glycerol replacement [26]. Moreover, wound healing is significantly impaired in these animals, with low keratinocyte proliferation, a feature that can also be reversed *in vivo* by feeding mice with glycerol [27]. Interestingly, inhibition of AQP3 in keratinocyte cell cultures results in reduced water and glycerol permeability and impaired cell migration. The protein facilitates migration by functioning as a water channel, but is also implicated in epidermal cell proliferation as a glycerol transporter [27]. Consistent with this finding, mice lacking AQP3 expression not only display impaired epidermal cell proliferation but are also resistant to skin tumorigenesis [11]. This appears to be related to the ability of AQP3 to take up glycerol, a suitable energy substrate that supports cell growth. Nucleoside-derived drugs, particularly those used in antiviral therapy, may induce severe mitochondrial toxicity [28,29]. While this is not evident for nucleosides used in the treatment of solid tumors, recent evidence suggests that gemcitabine triggers moderate mitochondrial toxicity [30] and blocks the activity of human mitochondrial DNA polymerase [31]. Nucleoside derivatives additionally compete with intracellular nucleotides and inhibit key enzymes of the nucleoside salvage pathways [32,33], consequently impairing the cellular energy metabolism. In this context, it is feasible to assume that AQP3 induced after exposure to these drugs plays a compensatory role as a provider of energy substrates.

AQP3 silencing also reversed the up-regulation of selective p53-dependent transcriptional targets, such as the death receptor, FAS, implicated in apoptosis, and the inhibitor of the cyclin-CDK2 and -CDK4 complexes, p21, implicated in the modulation of cell cycle progression at G_1_. It is not clear from these observations whether AQP3 contributes to apoptosis in addition to its reported effect on cell cycle arrest, which is significantly reversed upon silencing of the gene. Interestingly, AQP3 itself is transcriptionally regulated by p73, a member of the p53 family, which exhibits similar biochemical properties but is rarely mutated in cancer cells [34]. p73 interacts with the transcriptional coactivator, Yes-associated protein (YAP), leading to enhanced p73-dependent apoptosis in response to DNA damage. YAP is stabilized by the product of the p73/YAP target gene, PML, under negative control by the proto-oncogenic AKT/PKB kinase [35]. Interestingly, the anticancer drug, curcumin, down-regulates AQP3 expression in cancer ovarian cells via a mechanism that involves, at least partially, inhibition of the EGFR pathway and downstream AKT [19]. While AQP3 is a p73 target, its association with pro-apoptotic processes does not appear relevant, at least under the conditions used here. This hypothesis is based on evidence that AQP3 up-regulation is observed only at 5-FU concentrations triggering cell cycle arrest, but not at higher doses in which cells are committed to programmed cell death. Moreover, the decrease in cell growth associated with short treatment with low doses of 5-FU is significantly reversed by knockdown of AQP3 expression. These observations collectively support the view that induction of this aquaglyceroporin is related to cell cycle arrest rather than apoptosis.

Aquaporins, including AQP3, are overexpressed in different tumors [12,13,15] and an oncogenic role was suggested for AQP5, although this protein is not an aquaglyceroporin [13,36]. To our knowledge, no correlation of basal or drug-induced AQP3 expression with drug chemoresistance has been reported to date. In view of the above findings, this issue deserves further investigation.

## Conclusions

In this contribution we addressed whether up-regulation of AQP3 following treatment with nucleoside-derived drugs, such as 5^′^DFUR and gemcitabine, is related to drug response. Experiments on MCF7 and HT29 cells with suppressed AQP3 expression confirm that this aquaglyceroporin is involved in the increase in cell volume following drug treatment and drug-induced cell cycle arrest. Thus, AQP3 up-regulation is not a collateral effect of nucleoside-derived drug action, but may be implicated in the ability of some cancer cells to respond to treatment.

## Abbreviations

5^′^-DFUR: 5^′^ deoxy-5-fluorouridine; AQP3: Aquaporin 3; 5-FU: 5-fluorouracil; hENT1: Human equilibrative nucleoside transporter 1; NBTI: Nitrobenzylthioinosine; AQP: Aquaporin/s; AQP1: Aquaporin 1; ER: Estrogen receptors; PR: Progesterone receptors; DMEM: Dulbecco’s Modified Eagle Medium; EC_75_: Effective concentration 75; MTT: 3-(4,5-dimethylthiazol-2-yl)-2,5-diphenyltetrazolium bromide; PCR: Polymerase chain reaction; CDKN1A/p21: Cyclin-dependent kinase inhibitor 1A; TNFRSF6/FAS: TNF receptor superfamily member 6; GAPDH: Glyceraldehyde 3-phosphate dehydrogenase; siRNA: Small interference RNA; FAM: 6-carboxy-fluorescein; PBS: Phosphate buffered saline; FITC: Fluorescein isothiocyanate; CuSO_4_: Copper sulphate; CDK2: Cyclin-dependent kinase 2; CDK4: Cyclin-dependent kinase 4; YAP: Yes-associated protein; PML: Promyelocytic leukemia; AKT/PKB: Protein kinase B; EGFR: Epidermal growth factor receptor; AQP5: Aquaporin 5; SE: Standard error.

## Competing interests

The authors declare that they have no competing interests.

## Authors’ contributions

LTM performed the experiments described in Figures 4, 5 and 6, contributed to the experiments shown in Figures 1, 2 and 3, and drafted and submitted the manuscript. SPT contributed to the experiments described in Figures 1, 2 and 3, and helped with the writing and revision of the manuscript. FJC helped with the study design and critically reviewed the manuscript. MMA and MPA are responsible for the study design, provided guidance, supervised the work and helped with the writing and revision of the manuscript. All authors read and approved the final manuscript.

## Pre-publication history

The pre-publication history for this paper can be accessed here:

http://www.biomedcentral.com/1471-2407/12/434/prepub
